# Does a Large Ear Type Wheat Variety Benefit More From Elevated CO_2_ Than That From Small Multiple Ear-Type in the Quantum Efficiency of PSII Photochemistry?

**DOI:** 10.3389/fpls.2021.697823

**Published:** 2021-07-20

**Authors:** Yuting Li, Xin Li, Yujie Li, Shu Zhuang, Yongxiang Feng, Erda Lin, Xue Han

**Affiliations:** ^1^Key Laboratory of Agro-environment and Climate Change of Agriculture Ministry, Institute of Environment and Sustainable Development in Agriculture, Chinese Academy of Agricultural Sciences, Beijing, China; ^2^Jiangsu Key Laboratory for Bioresources of Saline Soils, Jiangsu Provincial Key Laboratory of Coastal Bio-agriculture, Jiangsu Provincial Key Laboratory of Coastal Wetland Bioresources and Environmental Protection, Jiangsu Synthetic Innovation Center for Coastal Bio-agriculture, School of Wetlands, Yancheng Teachers University, Yancheng, China; ^3^Key Laboratory of Tea Quality and Safety Control, Ministry of Agriculture, Tea Research Institute, Chinese Academy of Agricultural Sciences, Hangzhou, China; ^4^College of Agronomy, Heilongjiang Bayi Agricultural University, Daqing, China

**Keywords:** elevated CO_2_, chlorophyll fluorescence, quantum efficiency, PSII photochemistry, winter wheat variety

## Abstract

Recently, several reports have suggested that the growth and grain yield of wheat are significantly influenced by high atmospheric carbon dioxide concentration (CO_2_) because of it photosynthesis enhancing effects. Moreover, it has been proposed that plants with large carbon sink size will benefit more from CO_2_ enrichment than those with small carbon sink size. However, this hypothesis is yet to be test in winter wheat plant. Therefore, the aim of this study was to examine the effect of elevated CO_2_ (eCO_2_) conditions on the quantum efficiency of photosystem II (PSII) photochemistry in large ear-type (cv. Shanhan 8675; greater ear C sink strength) and small multiple ear-type (cv. Early premium; greater vegetative C source strength) winter wheat varieties. The experiment was conducted in a free air CO_2_ enrichment (FACE) facility, and three de-excitation pathways of the primary reaction of PSII of flag leaf at the anthesis stage were evaluated under two CO_2_ concentrations (ambient [CO_2_], ∼415 μmol⋅mol^–1^, elevated [CO_2_], ∼550 μmol⋅mol^–1^) using a non-destructive technique of modulated chlorophyll fluorescence. Additionally, the grain yield of the two varieties was determined at maturity. Although elevated CO_2_ increased the quantum efficiency of PSII photochemistry (Φ_PSII_) of Shanhan 8675 (SH8675) flag leaves at the anthesis stage, the grain number per ear and 1,000-kernel weight were not significantly affected. In contrast, the Φ_PSII_ of early premium (ZYM) flag leaves was significantly lower than that of SH8675 flag leaves at the anthesis stage, which was caused by an increase in the regulatory non-photochemical energy dissipation quantum (Φ_NPQ_) of PSII, suggesting that light energy absorbed by PSII in ZYM flag leaf was largely dissipated as thermal energy. The findings of our study showed that although SH8675 flag leaves exhibited higher C sink strength and quantum efficiency of PSII photochemistry at the anthesis stage, these factors alone do not ensure improved grain yield under eCO_2_ conditions.

## Introduction

According to the IPCC (The Intergovernmental Panel on Climate Change) report, atmospheric CO_2_ concentration has been on an increase since the industrial revolution and is predicted to increase to 550 μmol⋅mol^–1^ in 2,050 and 1,020 μmol⋅mol^–1^ (RCP8.5) by the end of the century (Stocker et al., 2013; Dier et al., 2019). Atmospheric CO_2_ is an essential environmental factor necessary for photosynthesis, and it is commonly believed that photosynthesis is stimulated by elevated CO_2_ (eCO_2_) in C3 crops, because the ribulose-1,5-bisphosphate carboxylase-oxygenase (RuBisCO) is not substrate-saturated under the current ambient CO_2_ (aCO_2_) concentrations (Long et al., 2006; Aranjuelo et al., 2013). As one of the most important C_3_ food crop, wheat (*Triticum aestivum* L.) has been demonstrated to be highly sensitive to climatic and environmental variations (Misra and Chen, 2015; Pandey et al., 2018; Urban et al., 2018). Several studies have examined the effects of eCO_2_ on wheat photosynthesis; however, most of the studies focus on the dark phase of photosynthesis. Moreover, the effect of eCO_2_ on the primary reaction of photosystem II (PSII) in wheat is not fully understood. Primary reactions of photosystems mainly involve converting light energy into a primary form of chemical energy (Mathis and Rutherford, 1987). Effective photosynthesis involves optimum light absorption by the photosystem and the use of absorbed light quanta in subsequent oxygen-evolving reactions (Barber, 2016). Therefore, there is a need to examine the primary reaction of PSII in wheat photosynthetic organs under future eCO_2_ environments for sustainable wheat production.

Earlier studies on crop responses to elevated CO_2_ suggested significant genotypic variability in growth and yield (Ziska et al., 2012; Tausz et al., 2013; Tausz-Posch et al., 2015; Erice et al., 2019). The differences in light energy dissipative mechanisms between varieties in response to eCO_2_ might offer opportunities for the selection and breeding of high grain yield varieties for future production conditions. In cereals, it has been suggested that the source-sink relationship is a key factor for photosynthetic efficiency response to elevated CO_2_ (Uddling et al., 2008; Tausz et al., 2013). It has been proposed that plants are capable of avoiding photosynthetic downregulation because of their ability to increase C sink strength (Aranjuelo et al., 2009). It is of great interest to know how elevated CO_2_ will influence photosynthetic CO_2_ fixation, photoassimilates metabolism, and source-sink relationships in different varieties.

In wheat plants, photoassimilates accumulate mainly in the form of starch in the steams and in the form of sucrose in the flag-leaf before heading. After heading, the stored sugar is remobilized and transported to the ears, the new sink organs. The contribution of carbohydrate assimilated before anthesis to grain yield is in the range of 20∼40% of grain yield (Cock and Yoshida, 1972). However, little information has been reported on the carbon metabolism and allocation of photoassimilates in wheat varieties with different ear types and sizes under elevated CO_2_. Identifying wheat varieties that can permit full utilization of photosynthetic capacity is crucial for breeding high-photosynthesis potential varieties that are suitable for growth under elevated CO_2_ environments. Hence, the main objective of this study was to analyze the responses of large ear type and small multiple ear-type winter wheat varieties to elevated CO_2_ concentrations using modulated chlorophyll fluorescence detection technology.

The modulated chlorophyll fluorescence detection technology can rapidly capture fluorescence signals originating only from the plants and highly sensitive physiological responses to plant physiological status, particularly the responses of PSII activity to environmental changes (Feng et al., 2015; Goltsev et al., 2016; Banks, 2018; Osipova et al., 2019). Moreover, it can also identify the physiological conditions of plants at larger spatial and temporal scales (Zarco-Tejada et al., 2002). Additionally, chlorophyll fluorescence detection can explain the stepwise flow of energy through PSII from light absorption, dissipation, and electron transport for photochemical reactions (Kalaji et al., 2014). Therefore, in the present study, we adopted chlorophyll fluorescence detection technology to explore the effects of eCO_2_ on the quantum efficiency of PSII photochemistry in large ear and small multiple ear-type wheat varieties. The objectives of the study were: (i) to analyze the effect of eCO_2_ on chlorophyll fluorescence, photochemistry, and thermal dissipation in large ear and small multiple ear-type wheat varieties; (ii) to determine whether large ear type winter wheat variety with greater ear C sink strength (var. Shanhan 8675) possess higher quantum efficiency of PSII than that does the small multiple ear-type variety (cv. Early premium) under eCO_2_ environment; and (iii) to analyze the correlation between yield parameters and photosynthetic parameters, and to explore their responses to eCO_2_. The main hypothesis of this study is that the PSII primary photochemistry reaction of large-ear wheat variety responds positively (higher quantum efficiency of PSII photochemistry and lower non-photochemical energy dissipation quantum) to elevated CO_2_.

## Materials and Methods

### Experimental Site and Mini-FACE System

**FIGURE 1:**
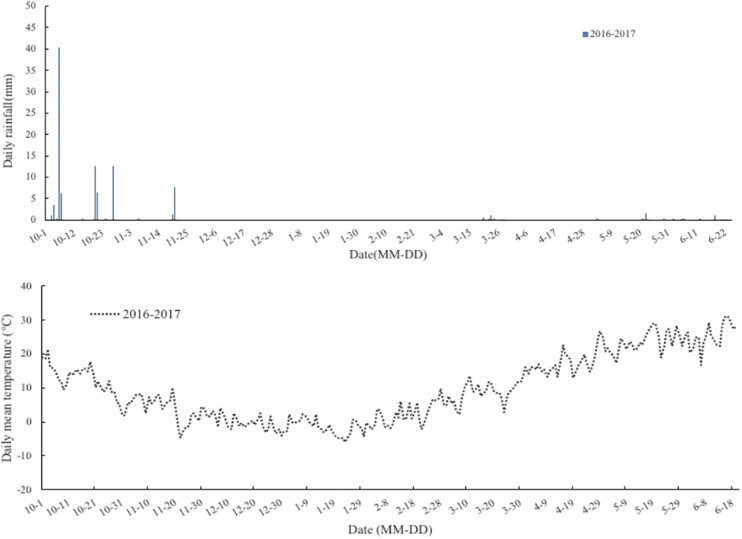
Rainfall (mm) and daily temperature (°C) at the wheat-maize rotation CAAS-FACE system in Changping, Beijing, China, from sowing of winter wheat until maturity during 2016–2017 experiment years.

**FIGURE 2:**
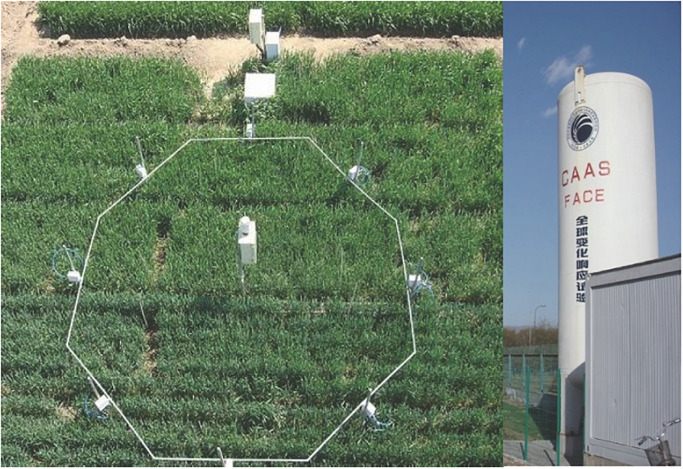
Mini-free air carbon dioxide enrichment system of Chinese Academy of Agricultural Sciences (CAAS-FACE system) in Changping, Beijing, China.

The experiment was conducted in a wheat-maize rotation mini-free air carbon dioxide enrichment system of Chinese Academy of Agricultural Sciences (CAAS-FACE system) in Changping (40°10′N, 116°14′E), Beijing, China, from 2016 to 2017. The soil (0–0.20 m) used for the study was a clay loam with pH (soil:water ratio of 1:5) of 8.4, organic C content of 14.10 g⋅kg^–1^, total N of 0.82 g⋅kg^–1^, available phosphorus of 19.97 mg⋅kg^–1^, and ammonium acetate extractable potassium of 79.77 mg⋅kg^–1^. The mean rainfall and temperature during the wheat growth period were 203 mm and 8.0°C, respectively (Figure 1). The Mini-FACE system consisted of 12 experimental plots, including six eCO_2_ rings (550 ± 17 μmol⋅mol^–1^) and six ambient CO_2_ (aCO_2_) rings (415 ± 16 μmol⋅mol^–1^), each with a diameter of 4 m. The experimental plots were at least 14 m apart to minimize cross-contamination of CO_2_ between the experimental treatments (Han et al., 2015). The CO_2_ enrichment treatment was accomplished using eight steel release pipes arranged like octagon corners, which released CO_2_ gas (Figure 2). In the case of eCO_2_ treatment, CO_2_ enrichment commenced 1 week after sowing and terminated at maturity. The CO_2_ concentration was maintained at 550 ± 17 μmol⋅mol^–1^ throughout the study period.

### Plant Material and Fertilization

Two winter wheat (*Triticum aestivum* L.) varieties, SH8675 and ZYM, were selected for this study. According to the ear traits and harvest index (HI), SH8675 is regarded as a large-ear variety, while ZYM is regarded as a small-ear variety (Table 1). The winter wheat varieties were sown in each of the CO_2_ treatment plots at the same time, with a plot area of 3.75 m^2^. The planting density of SH8675 and ZYM wheat was 333 plants per square meter and rows interval was 20 cm in elevated CO_2_ rings (∼550 μmol⋅mol^–1^) and ambient CO_2_ rings (∼415 μmol⋅mol^–1^), with three replicates per treatment. The varieties were planted randomly in each plot to minimize the effects of soil variation. Granular urea (N, 46%), diammonium phosphate (N:P_2_O_5_ 13:44%), and potassium chloride (K_2_O, 60%) were applied as basal fertilizers at the rates of 100 kg⋅hm^–2^, 165 kg⋅hm^–2^, and 90 kg⋅hm^–2^, respectively. At the jointing stage, granular urea was applied as side dressing at a rate of 100 kg⋅hm^–2^ on April 28, 2017. Irrigation was applied twice during the entire growing season of the winter wheat: the wintering irrigation at a rate of 750 m^3^⋅hm^–2^ on November 23, 2016, and spring irrigation at a rate of 750 m^3^⋅hm^–2^ was applied at the jointing stage after side dressing fertilization.

**TABLE 1:** Ear traits and the ratio of harvest index of SH8675 and ZYM wheat varieties.

Ear-type	Variety	Ear length (cm)	Grain number per ear	Grain weight per ear	Plant height (cm)	Ear number⋅m^–2^	HI
Large	SH8675	7.75 ± 0.09	26.87 ± 1.15	0.98 ± 0.03	60.13 ± 0.54	600.63 ± 13.06	0.40 ± 0.02
Small multiple	ZYM	7.07 ± 0.07	19.80 ± 0.48	0.63 ± 0.02	89.82 ± 1.09	651.83 ± 12.70	0.33 ± 0.01
ANOVA results		0.00*	0.00*	0.00*	0.00*	0.00*	0.02*

The values in this table are average values based on data from 2017 and 2018. ANOVA results with * indicate significance at P < 0.05.

### Chlorophyll Fluorescence Measurements

Chlorophyll fluorescence parameters were measured using a pulse amplitude modulation fluorometer (MINI-PAM, Heinz Walz, Germany). Chlorophyll fluorescence measurements were performed using intact flag leaves (three plants from each CO_2_ treatment) at 9:00–11:30 at half-way anthesis stage (DC 65) (Zadoks et al., 1974). Generally, SH8675 reaches anthesis (213 d) earlier than ZYM (216 d). However, in the present study, both varieties reached anthesis on the same day under CO_2_ and eCO_2_ conditions. The leaves were dark-adapted for 20 min with leaf clips to determine the ambient temperature fluorescence of dark-adapted leaf when all reaction centers are open and closed (Fo and Fm, respectively). Fo was measured under a weakly modulated measuring light (< 1μmol photons m^–2^s^–1^), and the leaves were immediately illuminated with an intense saturating pulse light (8,000 μmol photons m^–2^s^–1^, pulse time, 1s) to obtain Fm. The leaves were then light-adapted for 20 min, then turn on the actinic irradiation until the fluorescence reaches a steady state, the steady-state chlorophyll fluorescence (Fs) was measured, and Fm′ in the light-adapted state was estimated under saturated pulse light. According to previous studies, other parameters were calculated using the formulae given in Table 2.

**TABLE 2:** Legends and formulae for the calculation of chlorophyll fluorescence parameters.

Notation	Description	Formulae
Fo′	Minimal fluorescence during the light-adapted state	Fo′ = Fo/(Fv/Fm + Fo/Fm′)
ΔFv	Variable fluorescence quenching	ΔFv = Fm-Fs
ΔFv/Fo	Variable fluorescence quenching rate	ΔFv/Fo = (Fm-Fs)/Fo
Rfd	Variable fluorescence descent ratio	Rfd = ΔFv/Fs
Fv/Fo	Potential PSII efficiency	Fv/Fo = (Fm-Fo)/Fo
Fv/Fm	Maximum photochemical efficiency	Fv/Fm = (Fm-Fo)/Fm
Φ_PSII_	Quantum efficiency of PSII photochemistry	Φ_PSII_ = (Fm′-Fs)/Fm′
qP	Photochemical quenching coefficient	qP = 1-(F-Fo′)/(Fm′-Fo′)
qN	Non-photochemical quenching coefficient	qN = 1-(Fm′-Fo′)/(Fm-Fo)
Φ_NPQ_	Regulatory non-photochemical energy dissipation quantum	Φ_NPQ_ = F/Fm′-F/Fm
Φ_NO_	Non-regulated non-photochemical energy dissipation quantum yield	Φ_NO_ = F/Fm

*Source from Baker and Rosenqvist (2004), Kramer et al. (2004), Klughammer and Schreiber (2008).*

### NSC Measurement and Calculation

Non-structural carbohydrates (NSC) were extracted from plants at the anthesis stage. Leaf samples were placed in paper bags, deactivated at 150°C, and then dried at 80°C to a constant weight. The samples were ground and sieved through a 0.5 mm sieve. Sucrose and starch contents were measured using a resorcinol reagent and 3,5-dinitrosalicylic acid colorimetry reagent according to the procedures described by Wang et al. (2019). The sucrose and starch contents of the samples were determined spectrophotometrically using a multimode microplate reader (Infinite 200 PRO Nano Quant, Tecan, Switzerland). In this study, sugar and starch concentrations were estimated for NSC (Pan et al., 2011).

### Statistical Analysis

Statistical analysis of the data generated in this study was performed using SPSS 18.0 and EXCEL 2016. The experiment was arranged in a split-plot design with the plots arranged in randomized complete blocks; and the CO_2_ concentrations (ambient or elevated CO_2_) were the whole-plot treatment and the winter wheat varieties with different ear-types were the split-plot treatment. A general linear model was used to estimate the main effects of CO_2_ and variety, as well as their interactions. Analysis of variance (ANOVA) was used to test for statistical significance to determine the differences between treatment means. Mean values were compared using the least significant difference (LSD) test and the means were considered significant at *p* < 0.05.

## Results

### Chlorophyll Fluorescence Yield and Attenuation

There was a 24.7% decrease (*p* < 0.05) in the Fm′ of ZYM and a 14.0% decrease (*p* < 0.05) in the Fs of SH8675 under eCO_2_ condition (Table 3). However, there were no significant differences in the Fm′ and Fs of the two varieties under eCO_2_ condition (Table 3). There was a 14.8 and 15.4% decrease (*p* < 0.05) in the ΔFv and ΔFv/Fo ratio of ZYM, respectively, under eCO_2_ condition; moreover, the ΔFv/Fo ratio of ZYM was significantly lower (*p* < 0.05) than that of SH8675 under eCO_2_ condition (Table 3). There was a 10.9% increase (*p* < 0.05) in the Rfd of SH8675 under eCO_2_ condition (Table 3); however, there was no significant difference in the Rfd of the two varieties under eCO_2_ condition (Table 3).

**TABLE 3:** Effects of elevated CO_2_ on chlorophyll fluorescence emission and attenuation of two winter wheat varieties.

Variety	CO_2_	Fo	Fm	Fo′	Fm′	Fs	ΔFv	Rfd	ΔFv/Fo
SH8675	aCO_2_	293.22 ± 9.29	1573.2 ± 63.6	272.4 ± 5.1	1153.5 ± 79.5	491.8 ± 35.6	1077.8 ± 49.1	2.2 ± 0.1	3.7 ± 0.2
	eCO_2_	273.89 ± 9.14	1552.8 ± 53.0	257.7 ± 13.9	1284.1 ± 133.4	422.8 ± 33.8	1056.4 ± 40.1	2.4 ± 0.1	3.9 ± 0.0
ZYM	aCO_2_	296.78 ± 11.56	1453.4 ± 107.3	278.4 ± 15.1	1231.2 ± 8.5	467.1 ± 30.5	1143.3 ± 46.3	2.6 ± 0.0	3.9 ± 0.1
	eCO_2_	296.44 ± 4.52	1419.6 ± 50.9	264.9 ± 6.8	926.9 ± 47.6	408.9 ± 25.8	974.1 ± 21.7	2.5 ± 0.1	3.3 ± 0.1
ANOVA results	CO_2_	0.10	0.21	0.09	0.01*	0.00*	0.12	0.03*	0.74
	Variety	0.05	0.00*	0.46	0.04*	0.67	0.00*	0.00*	0.02*
	CO_2_ × Variety	0.47	0.88	0.98	0.00*	0.41	0.07	0.03*	0.04*
	LSD	29.78	163.55	–	322.58	–	155.83	0.40	0.57

*Significant effects of eCO_2_ are indicated by ^∗^ p < 0.05. Values are expressed as the mean ± standard error. aCO_2_, Ambient CO_2_; eCO_2_, Elevated CO_2_; LSD, least significant difference.*

### Chlorophyll Fluorescence Quenching Coefficients

**FIGURE 3:**
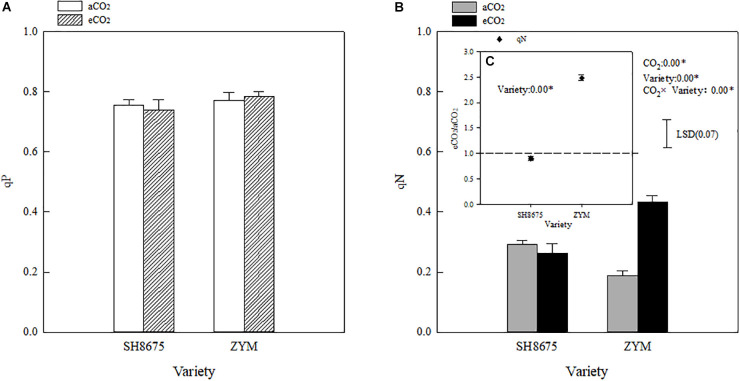
Effects of eCO_2_ on the qP and qN of two winter wheat varieties **(A–C)**. Measurements were carried out on intact flag leaves. Data represent the mean of three plants from each plot ± SD (standard error) bars. ANOVA results with * indicate significance at *p* < 0.05. Vertical bar in Figure 3B indicates LSD (*p* < 0.05) for qN. Differences in the qN of the varieties in response to eCO_2_ is showed in Figure 3C.

Elevated CO_2_ had no significant effect on the qP of the two winter wheat varieties; moreover, the effect of the varieties on qP was not significant (Figure 3A). However, eCO_2_ increased (*p* < 0.05) the qN of ZYM and SH8675 by 130.3 and 64.8%, respectively. Moreover, the qN of ZYM was significantly higher (*p* < 0.05) than that of SH8675 (Figures 3B,C), indicating that eCO_2_ significantly increased the thermal dissipation potential (more light energy absorbed by PSII was dissipated thermally) of ZYM compared with that of SH8675.

### Non-photochemical Excitation Energy Dissipation

**FIGURE 4:**
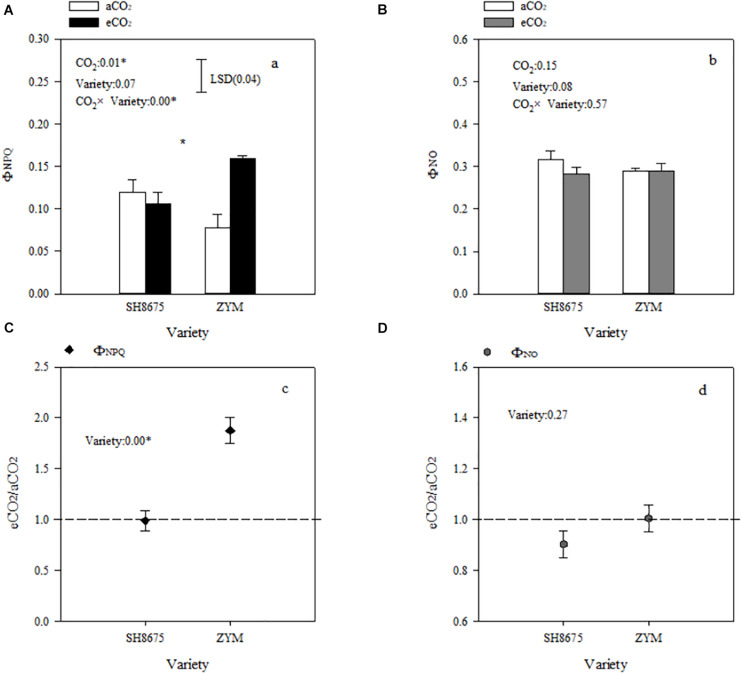
Effects of elevated CO_2_ on non-photochemical dissipation of two winter wheat varieties. Measurements were carried out on intact flag leaves. Data represent the mean of three plants from each variety plot ± SD (standard error) bars. ANOVA results with * indicate significance at *p* < 0.05. Vertical bars in **(A,B)** indicate LSD (*p* < 0.05) for Φ_NPQ_ and Φ_NO_, respectively. Differences of Φ_NPQ_ and Φ_NO_ between varieties in responses to eCO_2_ are shown in **(C,D)**, respectively.

Φ_NPQ_ and Φ_NO_ are positively related to light energy utilization in photochemical reactions. Elevated CO_2_ increased (*p* < 0.05) the Φ_NPQ_ of ZYM and SH8675 by 106.4 and 50.9%, respectively (Figure 4A). However, the Φ_NPQ_ of ZYM was significantly higher than that of SH8675 (Figure 4C), indicating that eCO_2_ significantly increased the thermal dissipation of ZYM, which resulted in lower quantum efficiency of PSII photochemistry. While elevated CO_2_ had no significant effect on the ΦNO of ZYM and SH8675 (Figures 4B,D).

### Photosynthetic Activity of PSII

**FIGURE 5:**
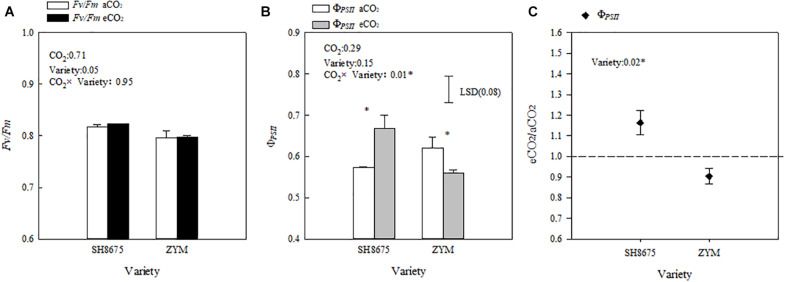
Effects of elevated CO_2_ on the Φ_PS__II_ and Fv/Fm ratios of two winter wheat varieties **(A–C)**. Measurements were carried out on intact flag leaves. Data represent the mean of three plants from each plot ± SD (standard error) bars. ANOVA results with * indicate significance at *p* < 0.05. Vertical bars in **(B,C)** indicate LSD (*P* < 0.05) for Φ_PS__II_ and Fv/Fo ratio, respectively. Differences of Φ_PS__II_ between varieties in responses to eCO_2_ are shown in **(C)**.

Elevated CO_2_ did not significantly affect the Fv/Fm ratio of the two varieties (Figure 5A). However, elevated CO_2_ increased the Φ_PSII_ of SH8675 by 16.3% (*p* < 0.05), but decreased that of ZYM by 9.9%.

### Carbohydrate Contents of Flag Leaves and Biomass at Anthesis Stage

For the large-ear variety, the levels of sucrose increased by 15.9% and the levels of starch in the flag leaves declined by 18.6% under elevated CO_2_ for SH8675. Contrast with the small multiple ear variety-ZYM, a larger amount of starch accumulated in the high-CO_2_ grown leaves than in the controls, while the sucrose contents were decreased by CO_2_ enrichment (Table 4). Additionally, there was a significant increase in the ear weight per unit area of the two varieties under eCO_2_ condition. However, the ear weight per unit area of SH8675 was 30.2% higher (*p* < 0.05) than that of ZYM (Table 4).

**TABLE 4:** Effects of elevated CO_2_ on agronomic characters of two wheat varieties at anthesis stage.

Variety	CO_2_ treatments	Leaves NSC		Leaves weight (g⋅m^–2^)	Ear weight (g⋅m^–2^)
		Sucrose content %	Starch content %		
SH8675	aCO_2_	39.12 ± 3.03	37.79 ± 2.60	148.74 ± 3.76	174.17 ± 5.83
	eCO_2_	45.34 ± 0.22*	30.76 ± 0.37*	167.66 ± 6.63	209.53 ± 6.49*
ZYM	aCO_2_	40.25 ± 0.29	35.52 ± 1.61	178.19 ± 4.00	188.34 ± 13.18
	eCO_2_	32.09 ± 1.57*	49.08 ± 2.32*	187.28 ± 11.98	160.94 ± 15.10*
ANOVA results	CO_2_	0.59	0.13	0.04*	0.64
	Variety	0.01*	0.00*	0.02*	0.07
	CO_2_ × Variety	0.00*	0.00*	0.81	0.01*
	LSD	5.45	6.72	–	4.17

Significant effects of eCO_2_ are indicated by * P < 0.05. aCO_2_: ambient CO_2_, eCO_2_: elevated CO_2_. LSD, least significant difference.

### Grain Number per Ear and 1,000-Kernel Weight

**FIGURE 6:**
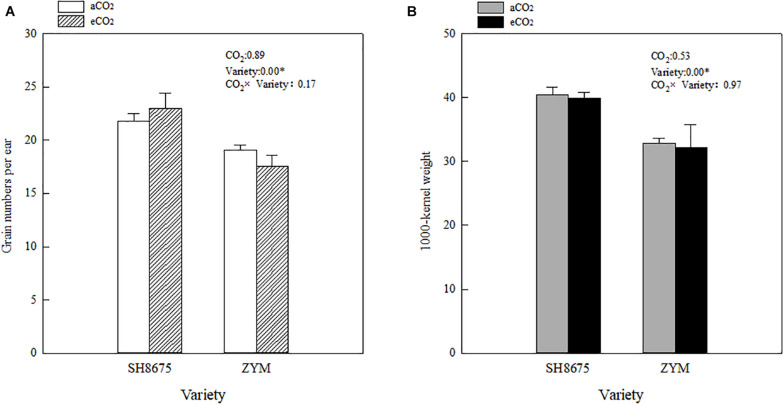
Effects of elevated CO_2_ on grain number per ear and 1,000-kernel weight of two winter wheat varieties **(A,B)**. Measurements were carried out at maturity stage. Data represent the mean of three plants from each plot ± SD (standard error) bars. ANOVA results with * indicate significance at *p* < 0.05.

There were no significant differences in the grain number per ear and 1,000-kernel weight between the varieties under eCO_2_ condition (Figure 6A). However, the grain number per ear of SH8675 was 31.1% higher (*p* < 0.05) than that of ZYM (increased by 16.3%) under eCO_2_ condition (Figure 6A). Although, aCO_2_ did not significantly affect grain numbers per ear and 1,000-kernel weight of the two varieties, SH8675 had a greater 1,000-kernel weight than that did ZYM under the two CO_2_ treatments (Figure 6B).

## Discussion

In the present study, we examined the effect of elevated CO_2_ on the primary reaction of PSII and carbon allocation in two winter wheat varieties with different ear C sink strengths. The results of the study showed that the greater ear C sink strength of SH8675 was beneficial for improved quantum efficiency of PSII photochemistry (Φ_PSII_) and the carbon allocation of the flag leaf under eCO_2_ at the anthesis stage. The carbohydrate content response to elevated CO_2_ varied in different ear type wheat varieties. For the high CO_2_ grown leaves, the starch content of SH8675 was significantly lower than that of the control, while that of ZYM was opposite. These results suggest that the high-CO_2_ grown leaves may function as stronger sinks for small multiple ear variety than the control leaves. It seems that excess carbohydrates produced by ZYM exposed to elevated CO_2_ and originally destined for storage in the stems and ears might be accumulated in the flag leaves, which are normally weak sinks.

**FIGURE 7:**
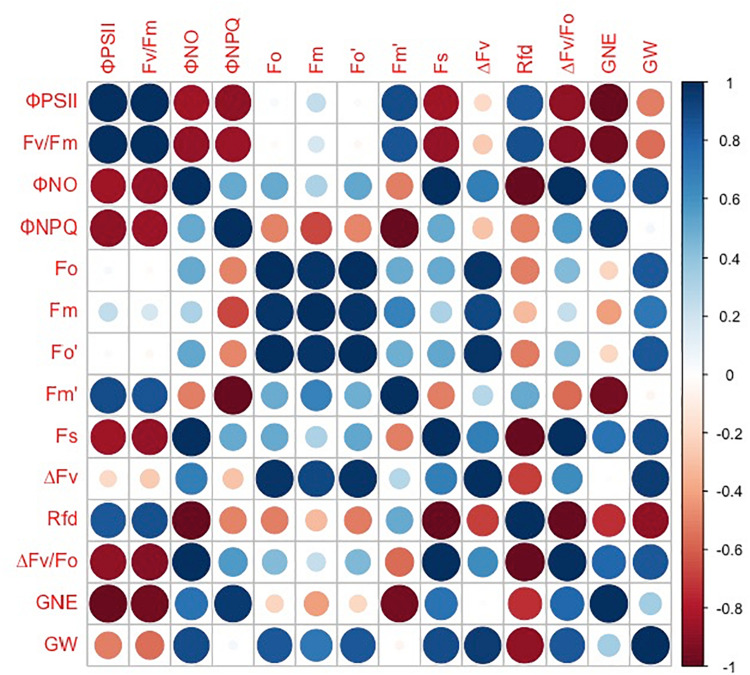
Correlations between grain number per ear (GNE), 1,000-kernel weight (GW), Φ_PSII_, Fv/Fm, Φ_NO_, Φ_NPQ_, Fo, Fm, Fo′, Fm′, Fs, ΔFv, Rfd, and ΔFv/Fo in SH8675. Spearman’s rank correlation coefficient-based correlograms of the measured parameters on plants grown under elevated CO_2_. The color of each square indicates the value of the correlation coefficient for each pair of traits following the color scale of the vertical color bar. The red and blue circles indicate negative or positive correlations between parameters, respectively.

**FIGURE 8:**
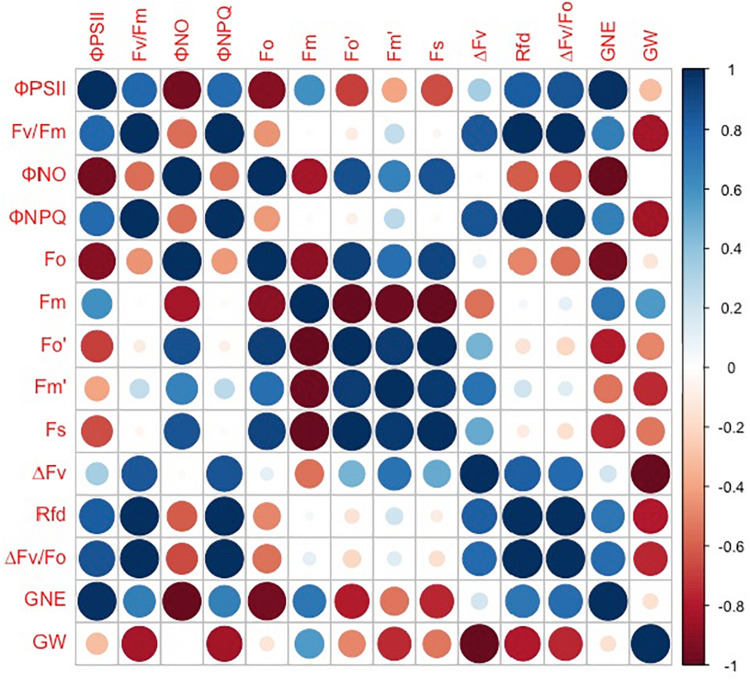
Correlations between grain number per ear (GNE), 1,000-kernel weight (GW), Φ_PSII_, Fv/Fm, Φ_NO_, Φ_NPQ_, Fo, Fm, Fo′, Fm′, Fs, ΔFv, Rfd, and ΔFv/Fo in ZYM. Spearman’s rank correlation coefficient-based correlograms of the measured parameters on plants grown under elevated CO_2_. The color of each square indicates the value of the correlation coefficient for each pair of traits following the color scale of the vertical color bar. The red and blue circles indicate negative or positive correlations between parameters, respectively.

However, the grain number per ear and 1,000-kernel weight of the wheat plants were not significantly affected by CO_2_ concentrations or varieties (Figure 6). In contrast, a lower Φ_PSII_ was observed in the flag leaf of ZYM at the anthesis stage, which was caused by an increase in the Φ_NPQ_ of PSII, suggesting that light energy absorbed by PSII in ZYM flag leaf was largely dissipated as thermal energy compared to that utilized for photochemical reaction. Furthermore, the results of correlation analysis showed that although eCO_2_ induced significant changes in the quantum efficiency of PSII photochemistry, these changes were not significantly correlated with grain number per ear and 1,000-kernel weight at the maturity stage in both wheat varieties (Figure 7). This result indicates that large ear type with high ear C sink strength alone does not necessary ensure effective utilization of eCO_2_ for grain yield. However, at the anthesis stage, ear C sink strength improved the quantum efficiency of PSII photochemistry of flag leaf in response to eCO_2_ condition (Figures 7, 8).

Light absorption by PSII is converted into energy, and most of the excitation energy is used for photosynthesis, a portion of the excitation energy is dissipated as heat, and a small percentage is emitted in the form of fluorescence (Maxwell and Johnson, 2000). Previous research has reported that elevated CO_2_ significantly increases PSII photochemical activity in cereal crops (Wang et al., 2015). Besides that, eCO_2_ led to decreases in both photorespiration rates and oxidative pressure was reported frequently (Leakey et al., 2009; Marçal et al., 2021). In the present study, the PSII photochemical activities of the two varieties in response to elevated CO_2_ were different (Figure 4C). There was a significant decrease in the Φ_PSII_ of ZYM under eCO_2_ condition (Figure 5B), which was caused by an increase in the qN of ZYM (Figure 3B), as indicated by the high Φ_NPQ_ of the flag leaves of ZYM (Figure 4A). Increased thermal dissipation in light-harvesting complexes competes with photochemistry for absorbed excitation energy, resulting in a decreased Φ_PSII_ (Yamamoto, 2016; Chen et al., 2018; Li et al., 2019). Therefore, it can be speculated that a large proportion of absorbed excitation energy of PSII in ZYM was dissipated as thermal energy, with lesser amount of energy used in photochemical processes. In contrast, eCO_2_ caused a 11.9 and 10.3% decrease in Φ_NPQ_ and Φ_NO_ of SH8675, respectively (Figures 4A,B); the Φ_PSII_ of SH8675 increased with increase in CO_2_ concentration from 415 μmol⋅mol^–1^ to 550 μmol⋅mol^–1^ (Figure 5B). Additionally, the Rfd value of SH8675 was significantly increased by eCO_2_, indicating that the potential photosynthetic activity of SH8675 was higher under eCO_2_ than that under CO_2_. Rfd is a vital indicator of the photosynthetic activity of plant leaf (Tuba et al., 1994), with a higher Rfd value indicating a higher photosynthetic rate (Lichtenthaler et al., 2005). Previous research has shown that plants increase non-photochemical quenching, with a down-regulation of PSII activity that causes a decrease in the photosynthetic carbon metabolism (Aljazairi et al., 2014; Mathobo et al., 2017). However, in this study, although eCO_2_ reduced the Φ_PSII_ of the flag leaf of ZYM at the anthesis stage, the grain number per ear and 1,000-kernel weight did not change significantly. Similarly, although eCO_2_ increased the Φ_PSII_ of the flag leaf of SH8675 at anthesis stage, the number per ear and 1,000-kernel weight were not significantly affected. These results lead us to ask if the responses of three de-excitation pathways to elevated CO_2_ differ due to different ear types in winter wheat varieties. The quantum efficiency of PSII photochemistry (Φ_PSII_) can be used to estimate the photosynthetic performance of the two varieties under both CO_2_ concentrations. In the present study, the wheat varieties were sensitive to eCO_2_. The Φ_PSII_ of SH8675 and ZYM were positively and negatively affected by elevated CO_2_, respectively. Hence, when CO_2_ increased to 550 μmol⋅mol^–1^, the PSII of SH8675 had a higher energy conversion efficiency than that did ZYM (Figures 5B,C). By analyzing the agronomic characteristics of these two winter wheat varieties, we found that the ear and leaf weights of SH8675 had the same response trend to elevated CO_2_ as that of Φ_PSII_ (Table 4). Additionally, eCO_2_ increased the sucrose ratio of the NSC of SH8675 flag leaf, but reduced the starch ratio (Table 4). Sucrose is the primary product of the source and substrate sink, and plays an important role in NSC metabolism and transfer into the ear (Griffiths et al., 2016; Weichert et al., 2017). Therefore, the above results indicated that under elevated CO_2_ condition, the flag leaf of the large-ear variety exhibited enhanced capacity for light energy utilization and an efficient translation of carbohydrates into the ear at the anthesis stage. Thus, efficient carbohydrate transport is important for the efficient utilization of light energy by winter wheat flag leaves, which is necessary for sustainable wheat farming under future climate change scenario. This is confirmed by the results of previous studies, which showed that sink-source imbalance can cause an accumulation of total non-structural carbohydrates (soluble sugar and starch) in source leaves, leading to a decrease in the photosynthetic capacity of leaves (Kasai, 2008; Daisuke et al., 2019). However, for the small multiple-ear variety, ZYM, a large quantity of energy absorbed by the flag leaves was largely dissipated as thermal energy, with limited amount being utilized for photochemical reaction under eCO_2_ condition. This could also be explained by the carbohydrate transfer theory, in which we analyzed NSC data and found that eCO_2_ increased the starch ratio of the NSC content of ZYM flag leaf, which can cause a decrease in the translocation of carbohydrates and subsequently, a decrease in ear weight (Table 4).

Furthermore, the effect of eCO_2_ on the ear weight of the two varieties at anthesis did not reflect in the grain number per ear and 1,000-kernel weight of the varieties at maturity stage. The reasons for this will be subject to further research. The methods need to be improved to explore the enzymatic activities of carbon metabolism and metabolites produced in photorespiration pathway, this is the limitations of the approach used in this study.

## Conclusion

In summary, the findings of the present study suggest that the high ear C sink strength of SH8675 improved the quantum efficiency of PSII photochemistry of the flag leaf in response to elevated CO_2_ and the translation of carbohydrates into the ear at the anthesis stage. In contrast, light energy absorbed by PSII in the ZYM flag leaf was largely dissipated as thermal energy, with relatively lesser amount being utilized for photochemical reaction; this resulted in a decrease in the translocation of carbohydrate to the ear and consequently a decrease in ear weight at the anthesis stage. However, the improvement in the quantum efficiency of PSII photochemistry of SH8675 flag leaf was not significantly correlated with grain number per ear and 1,000-kernel weight at maturity stage. Overall, the findings of our study indicate that high light utilization and high C sink strength alone does not necessarily ensure increased grain yield in wheat under eCO_2_ conditions.

## Data Availability Statement

The raw data supporting the conclusions of this article will be made available by the authors, without undue reservation.

## Author Contributions

YTL designed the study, performed the research and statistical analysis, and wrote the manuscript. SZ and YJL performed the experimental studies, data acquisition, and data analysis. XH conceived the idea and approved the final version of the manuscript. XL and EL contributed to conception and design of the study. YF provided intellectual content for this manuscript. All authors contributed to manuscript revision, read, and approved the submitted version.

## Conflict of Interest

The authors declare that the research was conducted in the absence of any commercial or financial relationships that could be construed as a potential conflict of interest.
